# Association of antidiabetic medication and statins with breast cancer incidence in women with type 2 diabetes

**DOI:** 10.1007/s10549-019-05185-0

**Published:** 2019-03-20

**Authors:** Mayu Hosio, Elina Urpilainen, Mikko Marttila, Ari Hautakoski, Martti Arffman, Reijo Sund, Ulla Puistola, Esa Läärä, Arja Jukkola, Peeter Karihtala

**Affiliations:** 1 Department of Oncology and Radiotherapy, Medical Research Center Oulu, Oulu University Hospital and University of Oulu, P.O. Box 50, 90029 Oulu, Finland; 2 Department of Obstetrics and Gynaecology, PEDEGO Research Unit, Medical Research Center Oulu, University of Oulu and University Hospital of Oulu, P.O. Box 23, 90029 Oulu, Finland; 3 Children, Adolescents and Families Unit, Department of Welfare, National Institute for Health and Welfare, P.O. Box 310, 90101 Oulu, Finland; 4 Orion Corporation, Orionintie 1, P.O. Box 65, 02101 Espoo, Finland; 5 Research Unit of Mathematical Sciences, University of Oulu, P.O. Box 3000, 90014 Oulu, Finland; 6 Service System Research Unit, National Institute for Health and Welfare, P.O. Box 30, 00271 Helsinki, Finland; 7 Institute of Clinical Medicine, University of Eastern Finland, P.O. Box 1627, 70211 Kuopio, Finland; 8 Department of Oncology and Radiotherapy, Tampere University Hospital, P.O. Box 2000, 33521 Tampere, Finland

**Keywords:** Breast cancer, Type 2 diabetes, Metformin, Statins, Cancer incidence, Cohort study

## Abstract

**Purpose:**

To address the possible association between the use of metformin, other forms of antidiabetic medication (ADM) and statins with the incidence of breast cancer in women with type 2 diabetes (T2D).

**Methods:**

Data were collected from a Finnish nationwide diabetes database (FinDM). The study cohort consisted of women diagnosed with T2D in 1996–2011 in Finland. In full-cohort analysis, Poisson regression was used to estimate hazard ratios (HRs) in relation to use of metformin, insulin, other forms of oral ADM and statins. In nested case–control analysis, up to 20 controls were matched for age and duration of diabetes to each case of breast cancer. Conditional logistic regression was used to estimate HRs in relation to medication use and cumulative use of different forms of ADM, and statins.

**Results:**

2300 women were diagnosed with breast cancer during follow-up. No difference in breast cancer incidence was observed between metformin users [HR 1.02, 95% confidence interval (CI) 0.93–1.11] or statin users (HR 0.97, 95% CI 0.89–1.05) compared with non-users. In nested case–control analysis the results were similar. Use of insulin (HR 1.18, 95% CI 1.03–1.36) was associated with a slightly increased incidence of breast cancer.

**Conclusions:**

No evidence of an association between the use of metformin or statins and the incidence of breast cancer in women with T2D was found. Among insulin users, a slightly higher incidence of breast cancer was observed.

**Electronic supplementary material:**

The online version of this article (10.1007/s10549-019-05185-0) contains supplementary material, which is available to authorized users.

## Introduction

Breast cancer is globally the most common cancer among females in both developed and developing countries [1]. Among all cancer deaths in women, 15% are caused by different types of breast cancer. Several risk factors of breast cancer have been identified, including age, early menarche, late menopause, nulliparity, use of oral contraceptives, hormone replacement therapy, family history and obesity [2]. People with type 2 diabetes (T2D) also have a higher risk of developing breast cancer [3, 4].

Metformin is commonly used as first-line oral medication for T2D. Over the years, it has been suggested that metformin may decrease breast cancer risk [5], but on the basis of a meta-analysis, there is insufficient evidence to support this hypothesis [6]. Many preclinical studies have shown anticancer effects of metformin in breast cancer and also in other types of cancer [7–9]. The insulin/insulin-like growth factor-1 signalling pathway and the adenosine mono-phosphate-activated protein kinase (AMPK) pathway are known to be related pathways involved in cancer growth. AMPK negatively regulates the mTOR (mammalian target of rapamycin) signalling pathway, resulting in inhibition of cancer proliferation and growth [7, 10]. The exact mechanisms are not yet known. Furthermore, it has been suggested that the use of glargine (a long-acting insulin analogue that is used in patients with diabetes) is associated with an increased incidence of breast cancer [11].

Statins, i.e. 3-hydroxy-3-methylglutaryl-coenzyme A (HMG-CoA) reductase inhibitors, are some of the most commonly used cholesterol-lowering drugs in the world. Reducing levels of mevalonate with statins are associated with apoptosis of cancer cells [12, 13]. Lipophilic statins seem to reduce tumour-cell proliferation and survival in vitro [14]. However, in a meta-analysis, the association between statin use and breast cancer incidence was not confirmed in the general population, although women with T2D were not assessed separately in this study [15].

In this register-based, cohort and nested case–control study, we enriched the evidence base concerning the association between the use of metformin, other types of ADM, and statins, with the incidence of breast cancer in women with T2D. By virtue of the large and comprehensive Finnish nationwide registers available to us, some of the pitfalls in previous studies could be overcome in this one.

## Patients and methods

In this article, we follow STROBE guidelines for reporting observational studies [16].

### Data sources and study population

A Finnish diabetes database (FinDM) has been created for epidemiological monitoring of diabetes in Finland [17]. This database consists of data on patients with diabetes, combining information from different registers in Finland: the Special Refund Entitlement Register and the Prescription Register, which are maintained by the Social Insurance Institution, The Care Register for Health Care and the Hospital Discharge Register from the National Institute for Health and Welfare, and the Causes of Death Register from Statistics Finland. The Special Refund Entitlement Register and the Prescription Register include information about purchases of ADM and statins since 1994, making it possible to accurately track the use of these medications. The Hospital Discharge Register and the Register for Health Care include diagnoses from hospital records since 1969 for inpatients, and since 1998 for outpatients, too. Patients with diabetes are entered in the register on the basis of diabetes diagnosis in hospital records or by receiving reimbursement for ADM. In some cases, the duration of diabetes may be longer than indicated in the register, as FinDM does not contain information on earlier diet-controlled diabetes followed in an outpatient primary-care setting. The classification of patients in the register to type 1 and type 2 diabetes was primarily based on the ADM used as first-line treatment. Comparison of data from FinDM against a local diabetes register covering the Helsinki region has shown good coverage of diabetic persons in the nationwide register [18].

The FinDM data were linked to the files of the Finnish Cancer Registry, which contain all cancer cases diagnosed in Finland since 1953 [19]. Data on mortality were obtained from Statistics Finland. Data linkage between various registers was carried out on the basis of the personal identification codes unique to each resident of Finland.

**Fig. 1:**
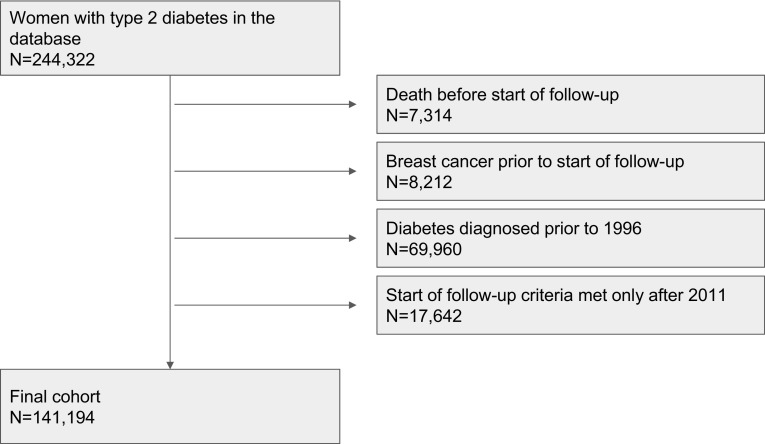
Flow chart showing how the cohort was found

The FinDM dataset contains data on approximately 240,000 women with T2D. In this study, we included women who were at least 40 years old and were newly diagnosed with T2D between the 1st of January 1996 and the 31st of December 2011. Women with prior breast cancer (diagnosed before the start of individual follow-up) and women with T2D who were diagnosed before 1996, and also women whose eligibility criteria would have been met only after 2011, were excluded. The final number of women with T2D in the cohort was about 140,000 (see Fig. 1).

Four indicators were defined for medication exposure: ever use of (1) metformin, (2) other types of oral ADM, (3) insulin and (4) statins. Details on Anatomical Therapeutic Chemical (ATC) codes of oral ADM and statins are listed in Supplementary Table. Exposure to a given medication was considered to begin a full year after its purchase date in order to avoid reverse causality problems. In the full-cohort analyses and in the nested case–control analyses, the study subjects were categorized as exposed to the drug from this moment onwards throughout the follow-up time. Additionally, cumulative use of metformin and statins, other forms of oral ADM and insulin were estimated by using defined daily doses (DDDs) purchased during the entire follow-up period (Jan. 1996–Dec. 2011).

Follow-up of each patient started 365 days after diagnosis of T2D. It ended on the date of breast cancer diagnosis, death, or the end of study period (the 31st of December 2011), whichever occurred earliest.

A nested case–control study within the cohort was conducted to evaluate the association between breast cancer and the cumulative use of the types of medication under study. This design, versus a full-cohort design, enables more straightforward calculation of the number of defined daily doses of medication used by each woman prior to their respective index date. For each case subject, up to 20 controls were selected without replacement from those women in the cohort who were alive and at risk of breast cancer at the date of breast cancer diagnosis of the case, and who were also matched for both age (date of birth ± 182 days) and duration of diabetes (± 182 days).

### Statistical analysis

In the full-cohort analysis, a Poisson regression model [20] was used to estimate hazard ratios (HRs) with 95% confidence intervals (95% CIs) of the incidence of breast cancer in relation to use of metformin, other forms of ADM and statins. In addition, the effects of current age and duration of T2D were assumed to obey a piecewise constant hazards pattern over chosen intervals of these two time scales. Age was split into 5-year intervals from 40 to 44 years to 85–89 years plus one more interval covering ages over 90 years, and duration of type 2 diabetes into intervals that are shown in Table 1. In the nested case–control analysis, conditional logistic regression [21] was utilized to estimate HRs with 95% CIs in relation to the use of different forms of ADM and statins. Cumulative doses were categorized according to tertiles of the total amounts of DDDs used. The register data were pre-processed using SAS/STAT software version 9.4 of the SAS System for Windows. Subsequent data transformations and statistical analysis were performed in R environment version 3.3.2 [22].

**Table 1:** Distribution of person-years at risk, numbers of cases and matched controls, and incidence rates of breast cancer by age, duration of diabetes and ever use of ADM and statins

Variable	Value	Person-years in cohort	Cases (%)	Controls (%)	Incidence (per 100,000 person-years)
Age (years)					
	40–49	48,365	59 (2.6)	1231 (2.7)	122.0
	50–59	134,663	346 (15.0)	6903 (15.1)	256.9
	60–69	205,392	715 (31.1)	14,229 (31.1)	348.1
	70–79	219,352	671 (29.2)	13,496 (29.5)	305.9
	80–89	141,150	448 (19.5)	8828 (19.3)	317.4
	90–106	19,739	61 (2.7)	1068 (2.3)	309.0
Duration of diabetes (years)					
	1–< 3	245,962	717 (31.2)	14,252 (31.1)	291.5
	3–< 5	180,562	522 (22.7)	10,290 (22.5)	289.1
	5–< 8	182,027	557 (24.2)	11,238 (24.6)	306.0
	8–< 16	160,082	504 (21.9)	9975 (21.8)	314.8
Metformin use					
	Ever	502,076	1514 (65.8)	30,588 (66.9)	301.5
	Never	266,557	786 (34.2)	15,167 (33.1)	294.9
Other oral antidiabetic medication use					
	Ever	376,233	1136 (49.4)	22,595 (49.4)	301.9
	Never	392,400	1164 (50.6)	23,160 (50.6)	296.6
Insulin use					
	Ever	90,162	304 (13.2)	5399 (11.8)	337.2
	Never	678,471	1996 (86.8)	40,356 (88.2)	294.2
No antidiabetic medication		145,612	412 (17.9)	8103 (17.7)	282.9
Statin use					
	Ever	384,679	1165 (50.7)	23,935 (52.3)	302.8
	Never	383,954	1135 (49.3)	21,820 (47.7)	295.6
Total		768,633	2300 (100)	45,755 (100)	299.2

## Results

Our final study cohort consisted of 141,194 women diagnosed with T2D between 1996 and 2011 and at least 365 days after diabetes diagnosis (Fig. 1). The total follow-up covered 768,633 person-years at risk (Table 1), the mean follow-up time being 5.4 years. During the study period, 2300 women were diagnosed with breast cancer for the first time (Table 1). Of these, 72% were ductal breast cancer cases and 17% had lobular histology.

The age-specific incidence rate (cases/100,000 person-years) of breast cancer was highest in the group of women aged 60–69 years (Table 1). The incidence of breast cancer was higher in patients whose duration of diabetes was over 8 years, compared with those with a shorter duration of the disease. Of the 2300 diabetic women diagnosed with breast cancer, 65.8% were metformin ever-users, and 49.4% were users of other types of oral ADM. A minority (13.2%) used insulin, and 17.9% had no ADM. Just over half of the patients (51%) were statin users, of whom 71% used simvastatin and 27% atorvastatin (Supplementary File 1).

In the full-cohort Poisson regression model (Table 2), no association was found between the incidence of breast cancer and use of metformin at any time (adjusted HR 1.02, 95% CI 0.93–1.11). However, use of insulin at any time was observed to be weakly associated with an increased incidence of breast cancer (HR 1.18, 95% CI 1.03–1.35). No association between the incidence of breast cancer and use of statins at any time was discerned (HR 0.97, 95% CI 0.89–1.05). The results of the nested case–control analysis were practically the same as regards use of the different types of medication under study (Table 2).

**Table 2:** Adjusted estimates of hazard ratios (HRs) with 95% confidence intervals (CIs) for the association between breast cancer incidence and ever use of metformin, insulin, other types of oral ADM, and statins compared with no use of these forms of medication at any time

Ever use	HR (u)	HR (a)	HR (c)
Metformin	1.02	1.02 (0.93–1.11)	0.94 (0.86–1.04)
Insulin	1.15	1.18 (1.03–1.35)	1.18 (1.03–1.36)
Other oral antidiabetic medication	1.02	0.95 (0.87–1.04)	0.98 (0.89–1.08)
Statin	1.02	0.97 (0.89–1.05)	0.93 (0.85–1.02)

*u* unadjusted, *a* adjusted, full-cohort Poisson regression, *c* adjusted, case–control conditional logistic regression

**Fig. 2:**
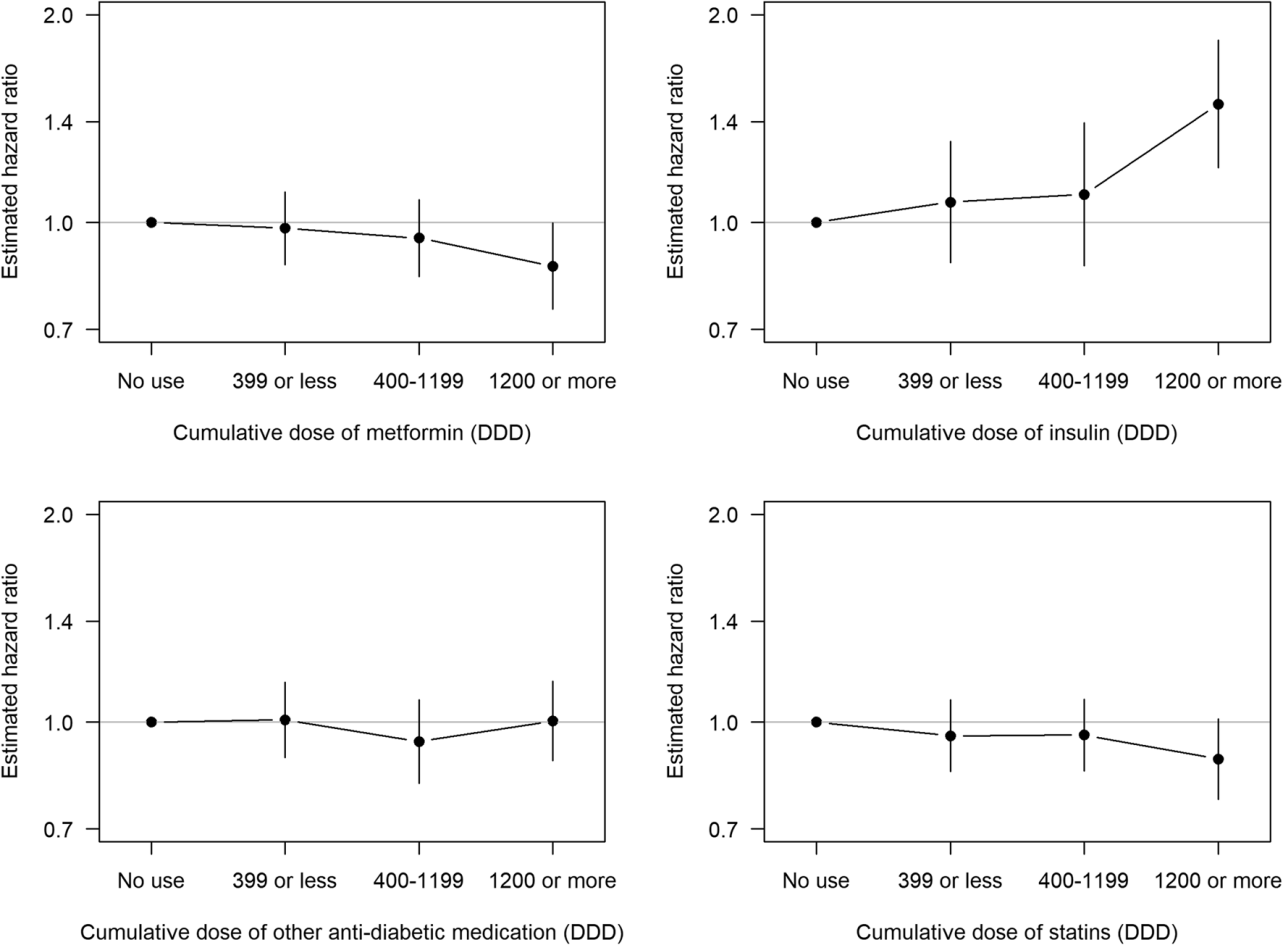
Estimated hazard ratios of breast cancer by cumulative doses of different forms of ADM and statins, adjusted for age, duration of diabetes and use of other medication

Apart from a slightly lower incidence among women whose cumulative DDD of metformin was at least 1200, there was no evidence of consistent dependence of the incidence of breast cancer on the cumulative use of metformin, other forms of oral ADM, or statins. A positive trend in breast cancer risk was observed with increasing cumulative use of insulin, the estimate being 1.46 (95% CI 1.19–1.81) for the amount of DDDs being 1200 or more when compared with no use at any time (Fig. 2).

There was no evidence of an interaction effect of metformin and statin use in the case–control analysis, the interaction HR being 1.03 (95% CI 0.86–1.24).

## Discussion

In our study, no evidence of any association between metformin use and the incidence of breast cancer in either the full-cohort analysis or in the case–control analysis was found in women with T2D. A similar result was observed in connection with use of other forms of ADM and statins. However, insulin use was found to be associated with a slightly increased incidence of breast cancer in the full cohort as well as in the case–control analysis. Furthermore, increasing cumulative use of insulin was observed to be positively associated with the risk of breast cancer in a monotonic manner.

Several epidemiological studies on breast cancer incidence and metformin use have been published before. In some of them, metformin use seems to be related to a lowered breast cancer incidence [23–26], at least in connection with long-term metformin use [27, 28]. However, similarly to our findings, most of these studies have not revealed any association between metformin use and the incidence of breast cancer [29–34]. In one study, the use of metformin was reported to be associated with an increased risk of breast cancer among insulin users [35]. In our study also, insulin use was associated with an increased incidence of breast cancer. Our findings are similar to those of some previous studies [36]. However, the increased risk of breast cancer has mainly been associated with long-term use of the insulin analogue glargine [11]. In our study, we did not analyse insulin types separately.

There are also several reviews and meta-analyses in which it has been concluded that statin use is not associated with the risk of breast cancer [37–42]. In a few studies, lipophilic statins, such as atorvastatin and simvastatin, have appeared to be associated with a decreased incidence of breast cancer [43]. In contrast to our study, the populations in these studies have not been limited to women with T2D.

Major strengths of our study are the large sample size and the availability of reliable and comprehensive national registers. The patients’ details are entered into the diabetes register at the time of the first reimbursement for any form of ADM, and thus the data in the register concerning the date of diagnosis of diabetes are considered to be accurate. To our knowledge, this study is the first one in which the association between statin use and the incidence of breast cancer in women with T2D has been explored. Also, our study has involved one of the largest study populations in addressing the relationship between ADM and breast cancer incidence.

A weakness of our study is that the FinDM database includes only information available in the registers. Therefore, we lack information on some known risk factors as regards the development of breast cancer, including body mass index (BMI), parity, use of contraceptives, use of hormone replacement therapy and family history. However, it is not known how these risk factors (apart from BMI) differ within ADM and statin users. Obesity is a common risk factor of both T2D and breast cancer. In the United Kingdom and the Netherlands, for example, metformin users have been found to have a higher BMI [44, 45]. Some review articles have described BMI as a time-dependent confounder [46]. Although the data on purchased medication are accurate, we could not cover the drugs dispensed in hospitals and outpatient clinics. However, only a small proportion of women with T2D are treated for long-term periods in healthcare facilities.

In conclusion, we found no association between metformin or statin use and the incidence of breast cancer in women with T2D. However, insulin use (especially cumulative use) was found to be associated with an increased incidence of breast cancer.

## Electronic supplementary material

Below is the link to the electronic supplementary material.

Supplementary material 1 (XLS 35 KB). Supplementary Table Details of Anatomical Therapeutic Chemical (ATC) codes and percentages of oral ADM and statins

## Abbreviations

ADM: Antidiabetic medication
AMPK: Adenosine mono-phosphate-activated protein kinase
ATC: Anatomical therapeutic chemical
BMI: Body mass index
CI: Confidence interval
DDD: Defined daily dose
FinDM: Finnish diabetes database
HMG-CoA: 3-hydroxy-3-methylglutaryl-coenzyme A
HR: Hazard ratio
ICD-O-3: International classification of diseases for oncology-3rd edition
mTOR: Mammalian target of rapamycin
T2D: Type 2 diabetes
